# Analysis of Land Use/Cover Change and Driving Forces in the Selenga River Basin

**DOI:** 10.3390/s22031041

**Published:** 2022-01-28

**Authors:** Yang Ren, Zehong Li, Jingnan Li, Yan Ding, Xinran Miao

**Affiliations:** 1 Institute of Geographic Sciences and Natural Resources Research, Chinese Academy of Sciences, Beijing 100101, China; reny.16s@igsnrr.ac.cn (Y.R.); lijn.17s@igsnrr.ac.cn (J.L.); miaoxinran@webmail.hzau.edu.cn (X.M.); 2 Faculty of Geography, Lomonosov Moscow State University, 119991 Moscow, Russia; 3 Department of Resources and Environment, University of Chinese Academy of Sciences, Beijing 100049, China; 4 Department of Geography and Ecotourism, Southwest Forestry University, Kunming 650224, China; doris_dy@yeah.net

**Keywords:** land use and land cover change, the Selenga River basin, driving mechanism, CA-Markov

## Abstract

The Selenga River basin is an important section of the Sino-Mongolian Economic Corridor. It is an important connecting piece of the Eurasian Continental Bridge and an important part of Northeast Asia. Against the background of the evolution of the geopolitical pattern since the disintegration of the Soviet Union and global warming, based on the land cover data in the Selenga River basin from 1992, 2000, 2009, and 2015, this paper describes the dynamic changes in land use in the basin. Through a logistic model, the driving factors of land cover change were revealed, and the CA-Markov model was used to predict the land cover pattern of 2027. The results showed that (1) from 1992 to 2015, the agricultural population in the Selenga River basin continued to decrease, which led to a reduction in agricultural sown area. The intensification of climate warming and drying had a significant impact on the spatial distribution of crops. Grassland expansion mostly occurred in areas with relatively abundant rainfall, low temperature, and low human activity. (2) The simulation results showed that, according to the current development trend, the construction land area of the Selenga River basin will be slightly expanded in 2027, the area of arable land and grassland will be slightly reduced, and the areas of forest, water/wetland, and bare land will remain stable. In the future, human activities in the basin will increase in the process of the construction of the China-Mongolia-Russia economic corridor. Coupled with global warming, the land/cover of the basin will be affected by both man-made and natural disturbances, and attention should be paid to the possible risk of vegetation degradation.

## 1. Introduction

Land use is the most direct and extensive human activity that changes the natural environment. Land use changes produce significant changes to the land surface and also have an impact on the material and energy flows of natural ecosystems, which in turn change the structure and function of ecosystems and affect ecosystem services and human survival and development [1]. In the process of global change research, land use change is the most closely intersecting issue between nature and humanity, and is a vivid manifestation of the human-earth relationship [2,3]. At present, land use change research is still one of the hot spots in the research of global change issues by scholars at home and abroad [4], and it is a key task to achieve the goal of sustainable development. The case studies in typical regions can grasp the characteristics of land use and cover change and scientifically explain the impact of human behavior on land cover and the interaction with ecological environment. The “Belt and Road” Ecological and Environmental Protection Cooperation Plan (2017) emphasizes that scientific research on land use is a new trend for future ecological protection cooperation among countries along the route to maintain the stability of regional ecosystems and human well-being.

For the relationship between driving forces and land use, quantitative research is mainly conducted by mathematical methods and analyzed by constructing models [5]. Among the typical methods of driving force research are: principal component analysis, typical correlation analysis methods, etc. The models are mainly system dynamics models, gray prediction models, Markov models, regression statistical models, etc. Logistic regression models can be applied with full consideration of whether the independent variables are continuous or not, and for categorical variables can also be added to the independent variable index system as factors. Moreover, the spatial heterogeneity of land type changes is taken into account, and the spatial driving factors are comprehensively covered, making the analysis process of driving mechanism more comprehensive and objective. Therefore, Logistic model is chosen in the analysis process of driving mechanism in this paper.

LUCC models need to be gradually developed and applied while continuously improving model functions and building an integrated and complete theoretical system to better realize simulation and prediction. Integrated models usually combine two or more modeling techniques, which can consider both spatial characteristics of land use change and non-spatial quantitative change characteristics. The CA-Markov model combines the Markov model’s focus on the quantitative land use change simulation and the CA model’s sensitivity to the spatial characteristics, so that the model can simulate the land use change process The CA-Markov model is applicable to the watershed scale and combines the relationship between land change and drivers to simulate and predict the spatial and temporal patterns of future land use types with high simulation accuracy, which is suitable for long-term prediction.

There are few studies on LUCC and dynamic analysis in the Selenga River basin. The literature search found that international articles on the study of the Selenga River basin are mostly directed at the physical properties, geochemical characteristics, and water resources of the basin. Wang Juanle [6,7] conducted a surface analysis of land-use change in Mongolia but did not quantitatively analyze its change mechanism. For the study of land change and landscape ecological impact in the outer area of Baikal and its typical cities [8], based on a surface analysis of the land-use patterns and changes in the outer Baikal region, the intrinsic driving mechanism and typical urban contrast landscape ecology are detailed. The results reveal the characteristics of socioeconomic development in the region from a unique perspective. The analysis of land-use changes and driving forces in the China-Mongolia-Russia cross-border region is limited, and there is room for further analysis and discussion. Therefore, based on remote sensing technology, combined with natural and socioeconomic data, this paper analyzes the spatial and temporal characteristics of LUCC and the driving forces, and provides a scientific basis for ecosystem protection in cross-border basin areas.

## 2. Case Area Overview and Method

### 2.1. Overview of the Study Area

Lake Baikal, located in the Buryatia Republic of Siberia, Russia, is the oldest and deepest lake on Earth and is known for its rich freshwater resources and biodiversity [9]. The water system in the Buryatia Republic is well developed, with many rivers flowing into Lake Baikal, and the Selenga River is one of its most important rivers [10].

**Figure 1:**
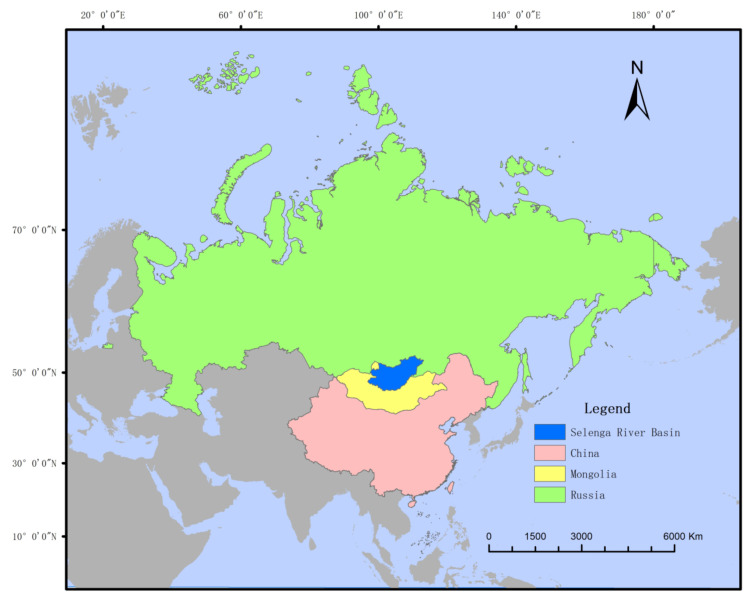
The location of the Selenga River basin.

The Selenga River originates from the northern slope of Mount Hangai in Mongolia, flows through northern Mongolia and east-central Russia, and flows into Lake Baikal, Russia, replenishing more than 60% of the lake’s water volume. The Selenga River forms the world’s largest lake delta with an area of nearly 700 km^2^ at the entrance of the lake [11]. As the largest tributary of Lake Baikal, it has a total length of 1024 km and a drainage area of 447,060 km^2^, accounting for 82% of the area of Lake Baikal. In recent years, the amount of water in the Selenga River basin has decreased, and the water level of Lake Baikal has dropped, causing increasing concern about the water environment. As the main upstream river and water source of Lake Baikal, the Selenga River basin is an area that is sensitive to global change, and it is a node of the Sino-Mongolian Economic Corridor, the Russian Eurasian Economic Union, the Mongolian Prairie Road, and the Sino-Mongolian crossborder high-speed rail corridor (Figure 1). The area overlaps with major constructive strategies among major powers and has unique politics and a unique economic geostrategic status. The ecological environmental pattern in this region is complex and diverse, and the interaction between natural processes and human activities has a profound impact on Northeast Asia’s resources, environment and social and economic development. Land use/cover change (LUCC) directly affects the water resource changes in the basin and affects the ecological security of Lake Baikal.

The Selenge River basin plays an important role in the socioeconomic life of Mongolia, with 19.2% of the total area of Mongolia [12], including the capital city of UlaanBator, and the second and third largest cities (Dalhan and Erdenet), where more than 60% and 80% of agricultural and industrial products, respectively, are produced in the Selenge river basin [13]. After the collapse of the Soviet Union, the Russian population decreased significantly, with the westward migration of the population from Siberia and the Far East and the migration of a large number of rural laborers, especially young ones, to the cities. Together with policies such as land privatization and collective farm reorganization, there was a significant abandonment of agricultural land and urban expansion in Siberia, resulting in a significant shift in the original land use patterns and trends. Land use/cover change studies are conducted in this watershed to analyze the processes and mechanisms of land change for sustainable watershed development.

### 2.2. Methods and Data

#### 2.2.1. Data

The land cover data of the Selenga River basin were extracted from the global land cover products. The data in 1992 were extracted from the UMD Land Cover data set, with a spatial resolution of 1 km and a temporal resolution of year 1992–1993; the data extracted for 2000 were from the Global Land Cover 2000 data set, the spatial resolution was 1 km; the 2009 data were extracted from the GlobCover data set; the 2015 data were extracted from FROM-GLC 2015_v1 download web URL data set.

The monthly average temperature and precipitation data from 1992 to 2015 were obtained from the Climatic Research Unit (CRU) of the University of East Anglia, with a spatial resolution of 0.5°.

The 90 m resolution SRTM DEM data set (Shuttle Radar Topography Mission Digital Elevation Model) was obtained from the Consortium for Spatial Information. The data came from the Global 3D graphical data project, jointly established by the National Aeronautics and Space Administration (NASA) and the National Geospatial-intelligence Agency (NGA), and we had access to 1:100,000 terrain and other raster data, administrative divisions, water systems, roads, and other vector data.

#### 2.2.2. Methods

1. Unifying the land use/cover classification of the Selenga River basin

In view of the different classifications adopted for the different phases of land cover products in the Selenga River basin required for the study, to meet the research objectives and based on the retrieved datasets, a new classification scheme with nine categories (Table 1) was proposed, comparison of different classification systems as shown in the Table 2 [14,15].

2. ANUSPLIN interpolation

Anusplin, a surface fitting software for climate data written by Australian scientist Hutchinson based on the theory of thin plate smoothing splines [16], is a professional software suitable for spatial interpolation of long-term meteorological elements [17]. This research used software to interpolate the meteorological driving force factors (temperature and precipitation) in the land-use change of the watershed to satisfy the driving force analysis process.

3. Logistic regression model construction

Using SPSS 24.0 software, this paper quantitatively studied the relationship between land-use types and driving factors by binary logistic regression analysis and obtained the regression coefficients of land-use types.

We took the changes (expansion or contraction) in the main land types (croplands, needleleaf forest, grassland) as the dependent variable, with the value “1” (indicating sample points where land use/cover changes have occurred), “0” (indicating sample points where no land use changes have occurred). Assign a value of “1” to the sample point of the gain of the land type, and assign a value of “0” to the sample point that has not changed and loss, and analyze the main driving factors of land type expansion; conversely, the loss of the land type is assigned a value of “1”, and the unchanged and gain value is assigned a value of “0” to analyze the main driving factors of land type contraction. Input the dependent variable and continuous and categorical independent variables into SPSS. After standardizing the continuous independent variable data, we used the logistic model in SPSS to perform regression analysis on the standardized continuous independent variables, categorical independent variables, and dependent variables to obtain the driving force for changes in main land types from 1992–2000, 2000–2009, and 2009–2015 and perform corresponding analysis. We used the HL (Hosmer and Lemeshow test) index as the goodness-of-fit index to test the model. According to the test result, using the significance level of >0.05, all passed the test, which meant the results were credible.

(1) Create a land-use change layer

**Figure 2:**
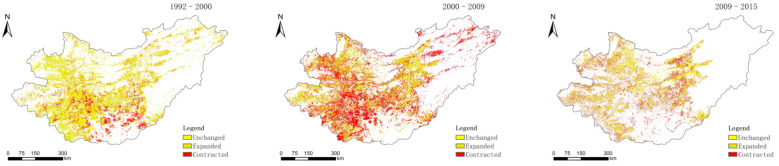
Distribution of cultivated land changes in 1992–2000, 2000–2009, and 2009–2015.

**Figure 3:**
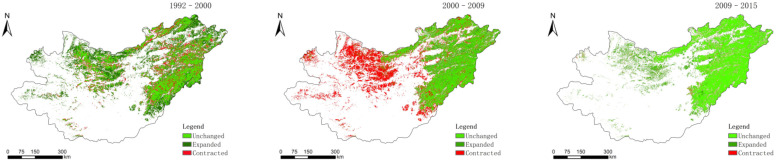
Distribution of coniferous forest land changes in 1992–2000, 2000–2009, and 2009–2015.

**Figure 4:**
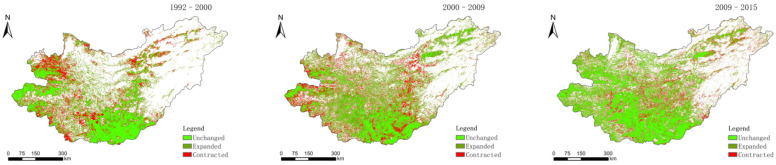
Distribution of grassland changes in 1992–2000, 2000–2009, and 2009–2015.

Three land-use change layers (1992–2000, 2000–2009, and 2009–2015) were made from cultivated land, coniferous forest land and grassland (Figure 2, Figure 3 and Figure 4).

(2) Establish driving factor layers

According to the principles of comprehensiveness, representativeness, accessibility, and regional difference in the driving force factors, combined with the natural environment and the social situation of the basin, nine influencing factors were selected from meteorology, society, space, and topography in this paper (Table 3).

**Figure 5:**
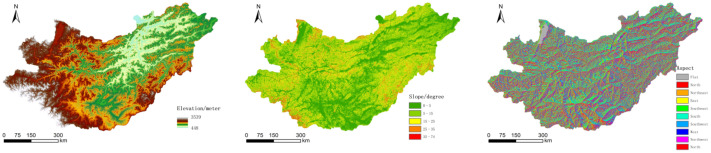
Elevation, slope, and aspect raster data in the basin.

This paper used 90-m DEM data and obtained the corresponding slope and aspect data through Spatial Analyst Tools/Surface/Slope and Aspect in ArcGIS (Figure 5).

**Figure 6:**
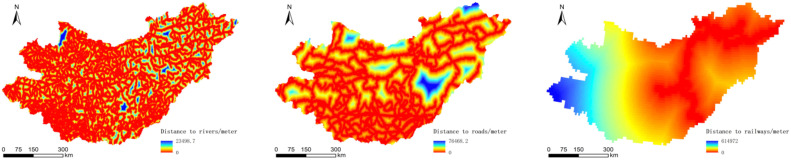
Raster data on distance to rivers, roads, railways.

The traffic neighborhood data were obtained by Spatial Analyst Tools/Distance/Euclidean Distance in the ArcGIS platform (Figure 6).

**Figure 7:**
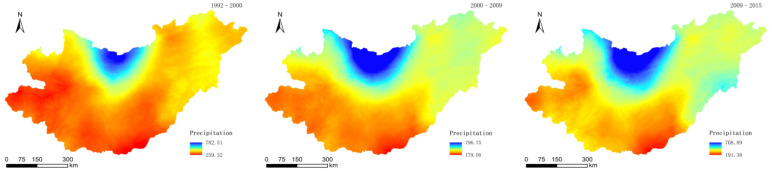
Precipitation in 1992–2000, 2000–2009, and 2009–2015.

**Figure 8:**
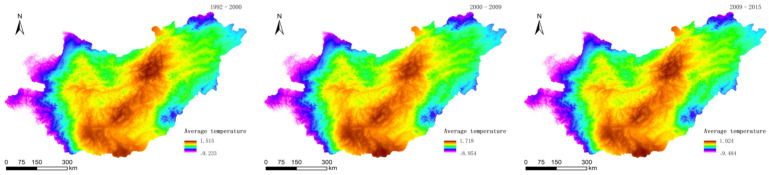
Average temperatures in 1992–2000, 2000–2009, and 2009–2015.

Through ANUSPLIN, the raster data layers of the precipitation (Figure 7) and average temperatures (Figure 8) in 1992–2000, 2000–2009, and 2009–2015 were obtained.

**Figure 9:**
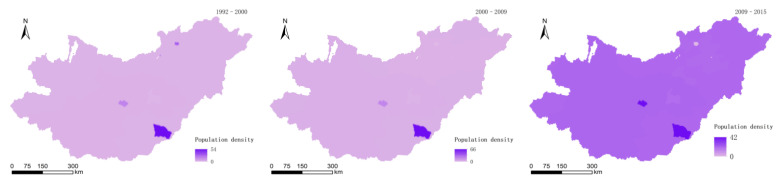
Population density changes in 1992–2000, 2000–2009, 2009–2015.

According to the official statistics of NASA, raster data of the world population in 1990, 2000, 2010, and 2015 were obtained, and raster layers of population density changes in the basin in 1992–2000, 2000–2009, and 2009–2015 were obtained (Figure 9).

The raster data obtained above needed to be standardized, that is, the projections needed to be uniformly converted into WGS-84, UTM49N, and the raster size was set to 100 m × 100 m.

(3) Random sampling and extraction of variable values

**Figure 10:**
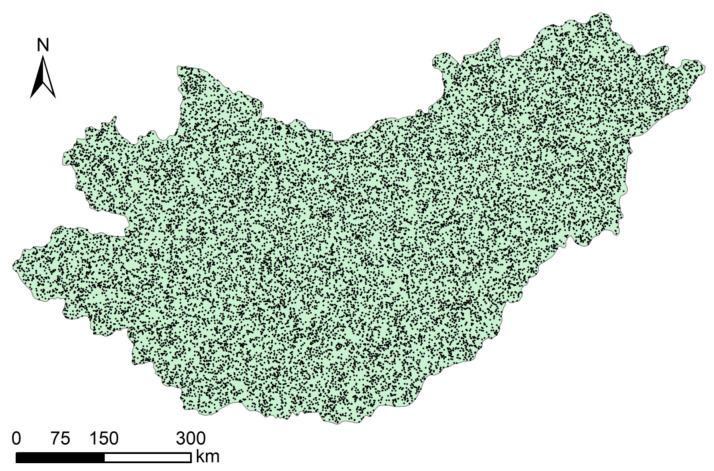
Random sampling points.

Through the pattern of random sampling, 20,000 sample data points uniformly distributed throughout the study area were selected (Figure 10).

(4) CA-Markov model construction

Using IDRISI Selva software, based on the land use distribution in 2000, combined with the land use type transfer matrix and suitability atlas from 2000 to 2009, the time span is 6 years to simulate and predict the land use distribution in 2015. The forecast results are compared with the actual land use distribution map in 2015. After the simulation accuracy is verified, the land use pattern in 2027 is simulated based on the land use in 2015 and the driving factors in 2009–2015.

(1) Cell composition

A 5 × 5 filter is used, that is, the 5 × 5 neighborhood cells around each central grid unit strongly act on the central cell and play a key role in its change.

(2) Make 2000–2009 (verification of prediction accuracy), 2009–2015 of each land type transfer area matrix and conditional probability matrix.
(3) Creating an atlas of land transfer suitability

Combined with the analysis of the driving forces of land use change, this paper will make conversion rules for construction land, cultivated land, coniferous forest land, grassland from the aspects of slope, aspect, distance to railway, distance to river, and distance to road to provide a basis for simulating the future land use distribution pattern of the watershed. The following main land types are obtained under the constraints of the suitability of each factor (Table 4), and make a suitability atlas through the suitability graphs of each category, which provides a basis for CA-Markov simulation prediction.

(4) Land use simulation and prediction

**Figure 11:**
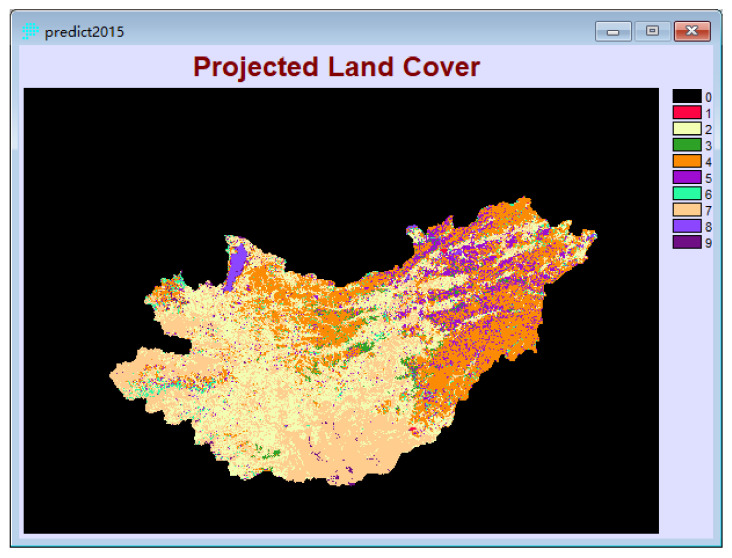
Land use Prediction in 2015.

Based on the land use distribution map in 2000, combined with the 2000–2009 land use type transition matrix and suitability atlas, the time span is six years, and the land use distribution in 2015 is simulated and predicted, and the prediction results (Figure 11) Compared with the actual land use distribution map in 2015, the Kappa coefficient is 0.76. Due to the long simulation period and changes in the objective natural environment and economic and social conditions, the simulation effect of this experiment is good and credible.

## 3. Results and Analysis

### 3.1. Land Use/Cover Pattern and Change

**Figure 12:**
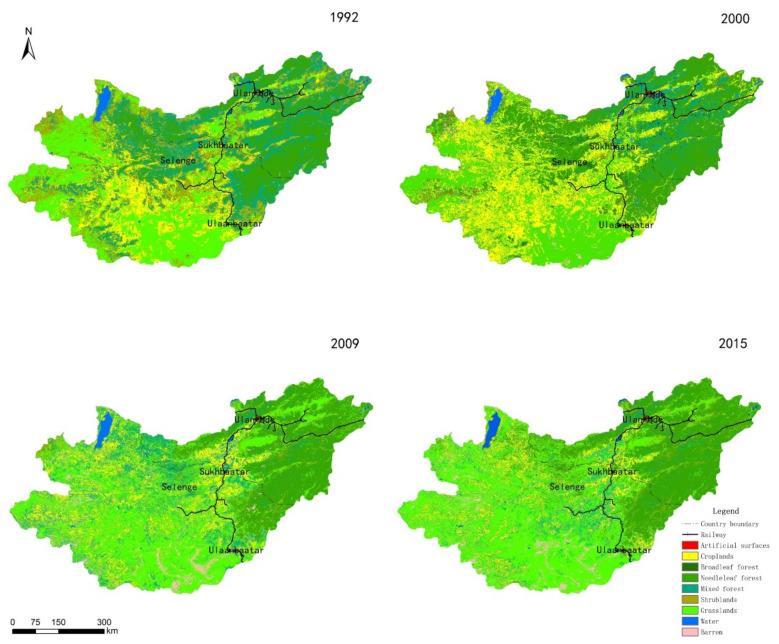
Land use/cover map of the Selenga River basin in 1992, 2000, 2009 and 2015.

From 1992 to 2015, the land use/cover pattern in the Selenga River basin (Figure 12) was mainly grassland and forest (coniferous forest, mixed forest, broad-leaved forest, shrubland). In 1992, these land types accounted for 28.4% and 59.33% of the total land area, respectively; in 2015, they accounted for 43.80% and 41.81%, respectively. These types are followed by cultivated land, water/wetland and barren, and the proportion of urban and rural construction land is relatively small.

**Figure 13:**
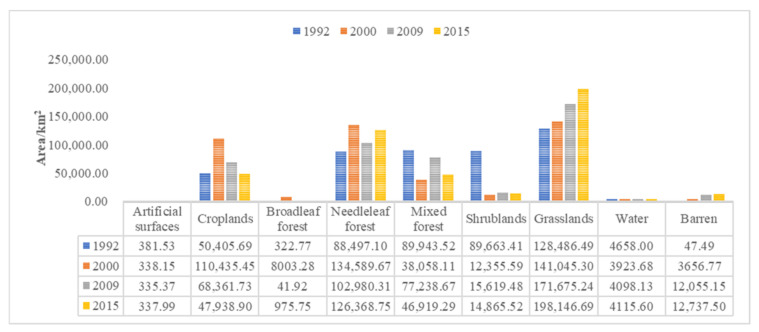
Classification and statistics of land use/cover patterns in the Selenga River basin.

From 1992 to 2000, the proportion of cultivated land and coniferous forest in the Selenga River basin increased from 11.14% and 19.56% to 24.41% and 29.75%, respectively; that of mixed forest and shrub decreased from 19.88% and 19.82% to 8.41% and 2.73%, respectively; the area of urban and rural construction land decreased slightly; and the area of broad-leaved forest increased slightly. From 2000 to 2009, the proportion of grassland, shrub and mixed forest increased to 37.95%, 3.45%, and 17.07%, respectively, the proportion of cultivated land and broad-leaved forest decreased to 15.11% and 0.11%, respectively, the area of coniferous forest decreased slightly, and the area of urban and rural construction land remained basically stable. Between 2009 and 2015, the proportion of cultivated land decreased to 10.60%, the area of forest land decreased slightly, and the grassland area increased to 43.80% (Figure 13).

Through the overlay analysis of land use data in 1992 and 2015, the land transfer matrix is obtained (Table 5). From 1992 to 2015, cropland was converted to forest and grassland, some of which were converted to barren; broadleaf forest, needleleaf forest, mixed forest, and shrubland were weakly converted to grassland at the same time; grassland was partially converted barren and forest; barren occupies a small proportion, which focuses on the transformation of grassland, forest, and cropland.

### 3.2. Changes and Distribution of Major Land Types

**Figure 14:**
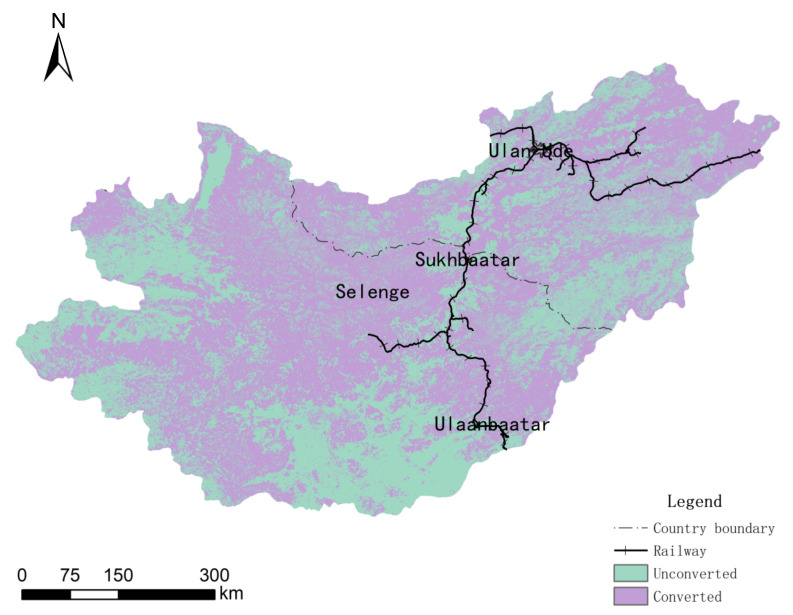
Distribution of transformed/untransformed plots in 1992–2015.

The land type conversion maps from 1992 to 2015 were superimposed and analyzed, and the plots that did not change were extracted. Such plots were mainly distributed in the central and western regions of the study area, with an area of 172,951.95 km^2^, accounting for 38.23% of the total area of the study area (Figure 14). The land use/cover changes in the Selenga River basin had obvious spatial differences in the past 23 years, and the land-use types of cities along the main railway lines in the river basin changed significantly. The eastern part of the basin, that is, the eastern part of the Russian Transbaikal, and the southern part of the basin, that is, the northern part of Mongolia, were relatively stable. The main railway lines from Russia to Mongolia had a direct impact on the land cover, and the conversion of various land-use types was relatively frequent; however, because the population densities of Russia and other areas of Mongolia are relatively small and human intervention is relatively weak, the land-use change in the region is relatively stable.

From 1992 to 2015, arable land changed significantly, and the conversion of cultivated land to grassland was mainly concentrated in western and central Mongolia and the southern Buryat Republic, Russia. There was also a certain degree of transformation along the railway. The conversion of cultivated land into forest land and bare land areas was mostly scattered in central and western Mongolia and Russia.

Broad-leaved forest, coniferous forest, mixed forest, and shrubland shifted, and the conversion of forestland to grassland was mainly distributed in northern Mongolia, while the conversion of forest land to bare land mainly occurred in western and central Mongolia. The conversion of shrubland to grassland mostly occurred in central Mongolia and along the railway; the conversion of mixed forest land to grassland was mostly concentrated in northern Mongolia and along the railway line; and the conversion of coniferous forest land to grassland was distributed in northern and eastern Mongolia.

**Figure 15:**
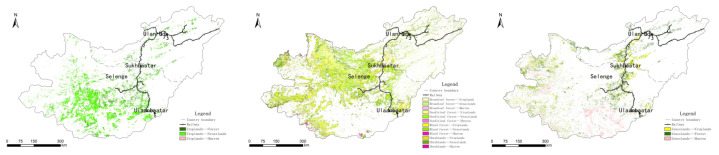
Cultivated land, forest land, and grassland conversion map of the Selenga River basin in 1992–2015.

The areas where grassland was converted to forest were mostly distributed in northwestern Mongolia and the central and southern regions of the Republic of Buryatia, Russia, with significant changes along the railway. In the city of Sukhbaatar in northern Mongolia, some grasslands were converted into cultivated land. In the central and northwestern parts of Mongolia, grassland was mostly transformed into bare land (Figure 15).

### 3.3. Analysis of Driving Factors of Major Land-Type Changes

According to the results of regression analysis (Table A1, Table A2, Table A3, Table A4, Table A5 and Table A6 in Appendix A), in the research stage, analyzing the contraction of croplands from natural factors, the declining precipitation and the rising temperatures had an important influence on the farmers’ decisions related to crop cultivation and the designation of development areas. Due to changes in natural conditions, the suitable area and environment for cultivation were changed, thus leading to a change in the area of croplands. In the later stage of the research, due to the deepening of human activities and the improvement of farming technology, farmers’ abilities to transform natural conditions improved, resulting in a decrease in precipitation and a decrease in cropland contraction probability. The effect of slope was not obvious, but in areas with higher elevations, the probability of cropland contraction went from increasing to decreasing. This pattern was because cropland contraction was more likely to occur in higher altitude areas where the natural environment was relatively unsuitable for crop growth. With the degree of human activity and the change in global climate, higher altitude areas were gradually developed for croplands; thus, the probability of contraction also decreased. Then, as the distance to the traffic lines decreased, the degree of human activity increased, and the more likely it was to be adversely affected. As distance from the rivers increased, there were fewer water sources and croplands were more likely to shrink. The higher the degree of population aggregation with the higher demand for reclamation was, the more severe the human disturbance to croplands was, and the greater the probability of contraction was.

With the decline in precipitation, the increase in temperatures and the decreasing probability of forest expansion, natural factors played an important role in the change in forest area, and their increase or decrease was closely related to natural conditions. Elevation and slope played major roles in forest expansion. The expansion of needleleaf forest land mainly occurred under topographic conditions with a slope of 5–15°, and its probability increased in higher altitude areas, which was closely related to the growth characteristics of needleleaf forests that were resistant to cold and drought. As distance from the traffic lines increased, the probability of forest land expansion increased. In addition, areas with lower population density had fewer human activities and weaker impacts on the natural environment, and the probability of needleleaf forest land expansion increased.

From the perspective of natural factors, precipitation, and average temperature played dominant roles in grassland expansion. Due to the unique climate environment in high elevation areas, the lower precipitation and the lower average temperature, the probability of grassland expansion decreased. Under topographic conditions with a slope of 5–15°, grassland was more likely to expand. The probability of grassland expansion in higher altitude areas first increased and then decreased, which had a certain relationship with the reduction in human activities in addition to the impact of the natural environment. The closer the distance to the rivers was, the more suitable the grass growth conditions were and the greater the probability of expansion was. In the later stage of the research, grassland expansion was more likely to occur in areas far from rivers because human activities were mostly concentrated in water sources. The farther the distance from roads and railways was, the weaker the influence of human activities was, and the probability of grassland expansion increased. Therefore, human activities had a great negative impact on grassland expansion.

### 3.4. Land Use/Cover Simulation and Prediction Analysis

**Figure 16:**
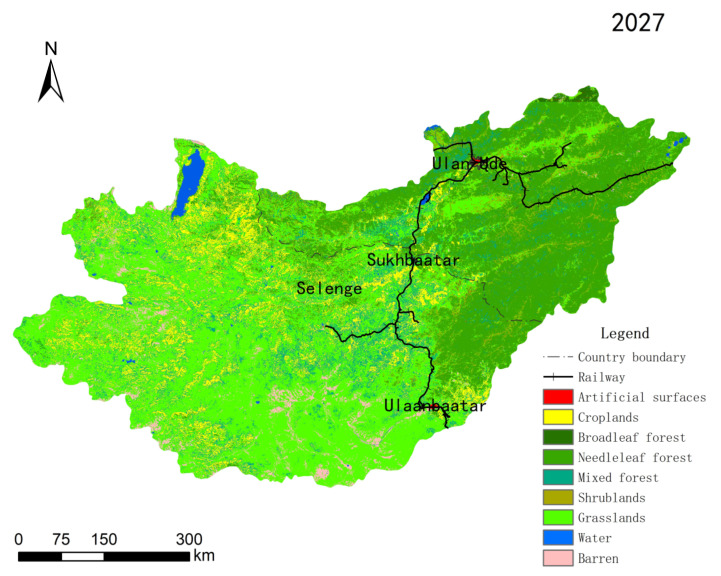
Land use prediction in the 2027 Selenga River basin.

Taking the land-use distribution data in 2015 as the base period, assuming that the land cover continues to develop according to the current law of change, using the 2009–2015 land-use transfer matrix and suitability atlas, we selected the interval year two times that was 2027, and we simulated and predicted the distribution pattern of land use in the basin in 2027 (Figure 16).

From the perspective of the spatial distribution pattern of land use in the Selenga River basin, the overall trend of land use was consistent. From the changes in land area, it was found that the area of construction land expanded slightly, the area of forest remained stable, and the area of grassland decreased. In terms of spatial distribution, the area of reduced croplands with obvious changes will mainly occur on the southern side of the Republic of Buryatia in Russia and the southwestern side of Mongolia, and a certain increase in cropland area will occur in the middle reaches of the basin. The area of forest will change more obviously along the main railway lines, mainly concentrated in the Republic of Buryatia in Russia and northeastern Mongolia. The change in grassland area will mainly occur in Mongolia, concentrated along the mainstream and tributaries of the upper and middle reaches of the Selenga River. The change and transfer of construction land area will mainly occur in the adjacent areas of cities in the two countries and around main railway lines. The water area will increase to a certain extent along the river system, mostly concentrated in the basin of the Selenga River in Russia into the Baikal Delta.

In future development, we should plan rationally and attach great importance to the characteristics of regional development. While rationally developing animal husbandry on the Mongolian side of the basin, we should also focus on improving the quality of grassland and achieving the improvement of both ecological and economic benefits. The abandonment of cultivated land on the Russian side has a profound impact on ensuring people’s production and living needs and stabilizing domestic food security. Therefore, in the process of the continuous deepening of urbanization and the continuous improvement of the economic level, the stability of cultivated land should be considered to better guarantee the area and quality of cultivated land. We should improve the level of basin development through more scientific and efficient planning and practices and provide support for the construction of important strategic nodes in the Silk Road Economic Belt and the China-Mongolia-Russia Economic Corridor.

## 4. Discussion

1. About the factors of land use change in the watershed. The transformation of various land-use types in the Selenga River basin over 23 years was closely related to the changes in local temperature and precipitation over time, and the indirect factors produced by human activities also had certain influences. The Selenga River Basin occupies only 22% of Mongolia’s total area, however, it contains between 55–60% of the nation’s population. Within the amigas in the basin, population densities range from a minuscule 1.3 to a massive 196.6 people per square kilometer. The obvious gap in population density fully reflects the differences in human settlements, and the basin still maintains the pattern of rural-to-urban migration. It is manifested in the gradual shrinking of small rural settlements, while the concentration of large urban settlements gradually increases. About 54% or Mongolians now live in urban areas, and more than 25% of the population live in the capital city [18].

With the increasing urbanization level in Mongolia [19], the population engaged in agriculture and animal husbandry continues to decrease. This further led to a reduction in cropland area, thus giving up a certain amount of agricultural land. Under severe changes in the natural environment, Mongolia in the basin has a high agricultural land use efficiency. The area of croplands in the basin has decreased since agricultural transformation, and the area of croplands in Mongolia in the basin has decreased by 27.5%. So far, there has been a trend of decreasing arable land. [20].

Although agriculture and livestock remained important to the Mongolian economy, state-directed economic planning onward increasingly emphasized developing Mongolia’s considerable and varied mineral resources. In addition to the migration and gathering to big cities, some industrial towns have appeared, linked by the Trans-Mongolian Railway to Ulaanbaatar as well as to Siberia. The development of new commodities (notably cashmere) and the establishment of mining ventures with foreign companies, tourism and other industries have become new channels for economic development [21].

The contraction of croplands was also driven by climatic factors such as reduced precipitation, which had an important influence on farmers’ decisions regarding crop cultivation and development areas. At the same time, climate disasters caused by climate change had a serious impact on crop harvest and cropland reclamation. Between 1999 and 2016, the area of farmland was further reduced by 25% [22].

2. Future development direction and cooperation. Despite the natural conditions, the intensity of development of the Mongolian food economy and the efficiency of farmland use in the agricultural economy is higher than in the Russian part of the basin. The efficiency of plant cultivation in Mongolia is positively influenced by the privatization of land and the dominance of agricultural organizations in land use. The existing structure of sown land in the Republic of Buryatia with a high proportion of fodder crops, satisfying livestock and bringing a small but stable profit for agribusiness. In the future, the development of new high-yielding drought-tolerant and early-maturing varieties will be the focus of development and then their introduction into the Buryat and Mongolian parts of the basin. To further increase the productivity of livestock and grazing livestock, there is a need to increase pasture production [20]. Land use change, which is currently more pronounced in the Mongolian than the Russian part of the Selenga River basin, is driven chiefly by mining and the expansion of agriculture [23,24]. The conversion of forests and natural grasslands into pastures and fields has implications for both hydrology [25] and water quality [26]. The resource cannot exist in isolation, and the integrity of the groundwater resource provides the primary source of water for urban centers, nomads and their livestock, agricultural lands, mining and tourism, and maintains the function of natural ecosystems. There are many contaminants that can endanger groundwater systems, such as increasing livestock access to streambeds leading to erosion problems and agricultural pollution, and direct discharges of manure and urine to surface waters. In addition, the relative inexperience of agricultural production may also lead to contaminants entering water sources, and both soil and water resources are negatively affected [18]. In the development process of the basin in the past few decades, more attention has been given to the ecological environment and water source protection of the basin. In the future, the basin will continue to warm, and the permafrost will continue to melt [27], which will cause profound changes in land use and water use. We should pay more attention to the important position of basin ecological strategies and improve the efficiency of agricultural land use. At the same time, to restore land cover, we recommend partially prohibiting grazing on degraded land and strictly controlling the implementation of environmental protection measures [28]. A more extensive program of integrated land and water resources management is needed in the future to gradually address these issues.

3. Sustainable Development under Policy Guidance. The “China-Mongolia-Russia Economic Corridor” is one of the six economic corridors of the “Belt and Road”, and its development and construction and related projects can, on the one hand, promote the advantageous production capacity cooperation of all parties, innovate the multilateral cooperation model, actively implement the development tasks under the leaders’ mechanism, and improve infrastructure construction and efficient economic cooperation. At the same time, it is also necessary to take into account local ecological sensitivities and constraints in the basin, to avoid nature reserves and harsh natural conditions as much as possible, and to proactively reduce the damage to the ecological environment caused by human activities. In this context, cooperation in the field of environmental protection is crucial, and the development of technologies and standards for the rational use of soil and water resources and pollution prevention, as well as permafrost protection, will minimize negative ecological impacts in order to achieve sustainable development [29]. The idea of sustainable land use was first introduced at the International Symposium on Sustainable Land Use Systems in 1990 and has gradually developed into a focus of land science research. The form and use of land will change at different times, and these changes may have certain negative effects on the ecological environment. In order to achieve the requirements of sustainability and adjust the internal structure to avoid damage to the limited land resources, it is necessary to coordinate the contradiction between ecological environment and social development in the process of land use, and finally achieve the coordination and sustainable development among population, resources, environment, and economy.

The concept of developing green economy has been agreed between China, Mongolia, and Russia, and sustainable development has become the main line of regional economic development. The type of land use and management should be configured in a rational way in order to maximize or optimize its ecological functions, production potential, and economic efficiency. It is not only to rely on high technology and good varieties, but more importantly to build a series of optimization models, such as optimizing the development model of regional land use in the construction of China-Mongolia-Russia economic corridor, actively developing low-carbon, and recycling economy, reducing the pressure of economic activities on ecological environment, reducing the impact of desertification and permafrost disasters, and achieving sustainable regional development. For Russia, its industrial structure is also homogeneous; Mongolia’s resource economy is more dependent. It should gradually find new dynamics of economic development in the development of regional economic cooperation and improve the level of open economic development; and it should unify the planning of the scope of ecological reserves, develop short-term and long-term protection plans, and establish ecological water connotation circles [30]. The implementation of the “North-South Water Transfer” project between Mongolia and Russia can be scientifically proven, and the joint ecological protection can completely eliminate desertification in the China-Mongolia-Russia Economic Corridor region; the indiscriminate cutting of forests, overgrazing, large-scale mining and other man-made activities are prohibited. Reasonable control of population growth in the region to avoid high population density. Reasonable use of water resources, water conservation, according to local conditions, to achieve a balance of water and soil. Adjust the structure of pastoralism and livestock grazing methods to improve the output rate and maintain land use stability; adopt seasonal pastures and avoid overgrazing [31]. Optimize the land use pattern, adjust the ratio of different land use types, rationalize development, and improve land use efficiency.

Close cooperation and collaboration are necessary among all stakeholders, including government agencies, communities, etc. Due to the uneven distribution of land and various resource uses within the watershed, many institutional improvements are needed. A multi-pronged approach is needed from macro and micro perspectives. At the international level, the Selenge River Basin is managed cooperatively by Mongolia and Russia for the benefit of both countries. Local-level management will complement the implementation of regional management strategies and promote efficient use of resources. There is also a need for educational activities for the public and general awareness of relevant issues such as resource conservation and wise exploitation. The cross-border basin ecosystem as a whole is in the same line as the neighboring countries and regions. Russia, Mongolia and neighboring countries should work together to protect the important ecosystems of forests, grasslands and wetlands in this region. This cross-border basin will become an important link of the “Belt and Road Initiatives” strategy and the construction of the China-Mongolia-Russia Economic Corridor, effectively enrich cross-border exchanges, broaden the direction of cooperation, become a new engine for development among countries, and promote cooperation between countries.

4. Deficiencies and Prospects. This paper introduces models to describe the relationship between land use changes and influencing factors in a time period, so as to reasonably adjust social and economic activities and scientifically utilize land resources. Because the problem of agricultural land abandonment is very complicated, it not only requires the analysis of objective images, but also the support and analysis of local actual first-hand data. It is a pity that this part has not been studied in depth and objectively due to objective factors. In the future scientific research, the agricultural land in this basin will also be analyzed in more depth. However, the research in this paper can not only grasp the changes of the Selenge River Basin in the time interval, but also provide methods and ideas for land use changes in other river basins. At the same time, the Selenga River Basin is a cross-border watershed, which involves different land resource conditions, different national backgrounds, and different lifestyles. The transboundary river basin ecosystem is a whole, and it is in the same line with the countries and regions along the route. The neighboring countries should act together to protect the ecological environment. Research in transboundary watersheds is conducive to comparative analysis of the ways and impacts of different countries on land resource use, and this impact can be amplified through long-term data accumulation. In order to grasp the changes of local land use and existing problems, scientifically explain the impact of human behavior on land cover and the interaction with the ecological environment. In order to better formulate development strategies, it can provide a useful reference for realizing the coordinated and sustainable development of the river basin.

In this study, the combination of manual visual interpretation and supervised classification is used for land change detection. AI, especially deep learning technology, has greatly improved the automation capability and accuracy of spatial information extraction from GIS, and has been playing an important role in several aspects, such as for remote sensing image feature extraction [32,33]. Therefore, in the future we will explore the application of AI algorithms in land classification research, not only to fully consider the complex land cover situation in different regions, but also to be able to carry out big data level land change monitoring and comparative analysis.

In the process of driving force analysis, there are also various methods and models, such as principal component analysis, which mainly selects several principal component factors instead of the original variables, thus reflecting most of the information; typical correlation analysis takes each group of variables as a whole, and quantitatively discriminates the degree of contribution through typical loading coefficients. In the future application process, suitable methods should be adopted according to the actual cases, sample size, and data accessibility.

In terms of model prediction, this paper focuses on the simulation of natural development scenarios, which is limited by the availability of data and therefore does not fully take into account the impact of policies. Therefore, an important direction for future research is to fully consider the two-sided effects of policies such as the China-Mongolia-Russia cooperation strategy. After obtaining the data related to the local ecological control line range and the planning strategy map, we can use them as the reference factors for the simulation, so that we can make simulation predictions for different scenarios, thus improving the simulation accuracy, making the land use pattern fitting results closer to the actual development situation, and better simulating the land use change development process.

## 5. Conclusions

In this paper, the Selenge River transboundary basin, which has an important geostrategic position, was selected as the study area. The global land cover dataset was used to obtain the 1992, 2000, 2009, and 2015 land use/cover change datasets of the basin to characterize the spatial and temporal dynamic patterns and processes of LUCC. Logistic models were used to model the driving mechanisms and to comprehensively analyze the driving forces of land use changes in the watershed from 1992 to 2015. The CA-Markov model was used to simulate and predict the future land use/cover distribution in the basin, with the aim of providing a scientific basis for the conservation and development of land resources in the cross-border region, and to better develop international land resource cooperation, prevent related ecological and environmental risks, and achieve sustainable development during the construction of the “One Belt, One Road” and the China-Mongolia-Russia Economic Corridor.

The main conclusions are:

1. From 1992 to 2015, the land-use types of the basin were mainly forest and grassland, plots of the land use/cover change were mainly distributed in the central and western regions of the research region, and the area of change accounted for 38.23% of the total area of the research region. The land use/cover change had obvious spatial differences, and the land-use transfer in the regions along the traffic lines was relatively severe. The area of construction land decreased slightly from 1992 to 2000, and maintained a generally stable trend from 2000 to 2015. The area of croplands increased from 1992 to 2000 and showed a decreasing trend from 2000 to 2015. The area of forest decreased during the study period, while the area of bare land and grassland kept increasing. The area of water/wetlands is basically unchanged.
2. Mathematical models were used to quantitatively and comprehensively characterize the effects of natural and social factors on land use changes. It was found that the transformation of each land use type in the Selenge River Basin over 23 years was closely related to the changes in local temperature and precipitation over the years, while indirect factors generated by human activities also had some influence. With the rising level of urbanization within Mongolia, the farming and herding population has been decreasing, leading to a decrease in arable land area. Other drivers of arable land shrinkage are climatic factors such as decreasing precipitation, which have a significant impact on farmers’ decisions on crop selection and development areas, and climatic disasters caused by climate change, which have a serious impact on crop harvesting and arable land reclamation. In addition, cropland shrinkage is preferred in higher altitude areas, but with the deepening of human activities and global climate change, these areas are also gradually used for cropland development, thus the probability of shrinkage decreases to a certain extent; due to the cold and drought tolerant growth characteristics of coniferous forests, area expansion is more likely to occur in areas with reduced precipitation, higher temperature, and higher altitude, while less human intervention also has a certain impact; in areas with abundant precipitation and lower temperature In the early stage, the closer the distance from rivers, the more likely the expansion of grassland will occur, while in the later stage of the study, the farther the distance from water systems and traffic arteries, the more likely the expansion of grassland in areas with inactive human activities.
3. The CA-Markov model was used to predict the land use/cover pattern of Selenge River Basin in 2027. From the change in the number of land types, it was found that the area of construction land expanded slightly, the area of forest land remained stable, and the area of grassland decreased. In terms of spatial distribution, the area of construction land around the cities in both countries increases slightly, the area of cultivated land increases somewhat in the middle reaches of the basin, but tends to shrink in the southwest side of Mongolia and the south side of Russia, the area of forest land expands in the northeast side, and the area of grassland expands significantly in the upper and middle reaches of the basin. There is a certain expansion of water area in the delta area of Selenge River.

## Figures and Tables

**Table 1:** Classification and Description of Land Use/Cover in the Selenga River Basin.

Class	Description
Artificial surfaces	Mainly include urban and rural areas, industrial and mining regions, transportation and other construction lands.
Croplands	Land mainly covered by crops that do not require irrigation or seasonal irrigation or crops that require periodic irrigation (mainly rice), including indistinguishable vegetation mosaic types containing farmland.
Broadleaf forest	Land covered by evergreen or seasonally deciduous broad-leaved trees.
Needleleaf forest	Land covered by evergreen or seasonally deciduous conifers.
Mixed forest	Land covered by broad-leaved and conifer trees with a coverage of 25–75% for each species.
Shrublands	Woody vegetation, height between 0.3–5 m.
Grasslands	Land covered by more than 15% herbaceous plants.
Water	Mainly include rivers, lakes, reservoirs, and areas that are periodically submerged by water.
Barren	Mainly refers to the surface almost no vegetation cover or vegetation is relatively sparse.

**Table 2:** Comparison of land use classification systems.

This Article Class Code	Umd 1992 Land Cover Data Set		GLCC 2000		Glob Cover 2009		FROM-GLC 2015	
	Code	Class	Code	Class	Code	Class	Code	Class
1	11	Croplands	17	Mosaic: Cropland/Tree Cover/Other natural vegetation	11	Post-flooding or irrigated croplands (or aquatic)	11	Rice paddy
			18	Mosaic: Cropland/Shrub and/or grass cover	14	Rainfed croplands	12	Greenhouse
			16	Cultivated and managed areas	20	Mosaic cropland (50–70%)/vegetation (grassland/shrubland/forest) (20–50%)	13	Other Cropland
							14	Orchard
							15	Bare farmland
2	14	Urban and Built-Up	22	Artificial surfaces and associated areas	190	Artificial surfaces and associated areas (Urban areas > 50%)	80	Impervious surface
3	2	Evergreen Broadleaf Forest	1	Tree Cover, broadleaved, evergreen	40	Closed to open (>15%) broadleaved evergreen or semi-deciduous forest (>5 m)	21	Broadleaf, leaf-on
	4	Deciduous Broadleaf Forest	2	Tree Cover, broadleaved, deciduous, closed	50	Closed (>40%) broadleaved deciduous forest (>5 m)	22	Broadleaf, leaf-off
			3	Tree Cover, broadleaved, deciduous, open	60	Open (15–40%) broadleaved deciduous forest/woodland (>5 m)		
					160	Closed to open (>15%) broadleaved forest regularly flooded (semi-permanently or temporarily)—Fresh or brackish water		
4	1	Evergreen Needleleaf Forest	4	Tree Cover, needle-leaved, evergreen	70	Closed (>40%) needleleaved evergreen forest (>5 m)	23	Needleleaf, leaf-on
	3	Deciduous Needleleaf Forest	5	Tree Cover, needle-leaved, deciduous	90	Open (15–40%) needleleaved deciduous or evergreen forest (>5 m)	24	Needleleaf, leaf-off
5	5	Mixed Forest	6	Tree Cover, mixed leaf type	30	Mosaic vegetation (grassland/shrubland/forest) (50–70%)/cropland (20–50%)	25	Mixed leaf, leaf-on
	6	Woodlands	7	Tree Cover, regularly flooded, fresh water	100	Closed to open (>15%) mixed broadleaved and needleleaved forest (>5 m)	26	Mixed leaf, leaf-off
			8	Tree Cover, regularly flooded, saline water				
			9	Mosaic: Tree Cover/Other natural vegetation				
			10	Tree Cover, burnt				
6	7	Wooded Grasslands/Shrublands	11	Shrub Cover, closed-open, evergreen	110	Mosaic forest or shrubland (50–70%)/grassland (20–50%)	41	Shrubland, leaf-on
	8	Closed Bushlands or Shrublands	12	Shrub Cover, closed-open, deciduous	130	Closed to open (>15%) (broadleaved or needleleaved, evergreen or deciduous) shrubland (<5 m)	42	Shrubland, leaf-off
	9	Open Shrublands	15	Regularly flooded shrub and/or herbaceous cover	170	Closed (>40%) broadleaved forest or shrubland permanently flooded—Saline or brackish water	71	Shrub and brush tundra
7	10	Grasslands	13	Herbaceous Cover, closed-open	120	Mosaic grassland (50–70%)/forest or shrubland (20–50%)	31	Pasture
			14	Sparse herbaceous or sparse shrub cover	140	Closed to open (>15%) herbaceous vegetation (grassland, savannas or lichens/mosses)	32	Natural grassland
					150	Sparse (<15%) vegetation	33	Grassland, leaf-off
					180	Closed to open (>15%) grassland or woody vegetation on regularly flooded or waterlogged soil—Fresh, brackish or saline water	72	Herbaceous tundra
8	0	Water	20	Water Bodies	210	Water bodies	51	Marshland
							52	Mudflat
							53	Marshland, leaf-off
							60	Water
9	12	Barren	19	Bare Areas	200	Bare areas	90	Bareland
			21	Snow and Ice	220	Permanent snow and ice	101	Snow
							102	Ice

**Table 3:** Driving factors system.

	Variable	Description	Unit	Source
Topography	Altitude	Elevation	m	DEM
	Slope	The degree of steepness of the surface unit	°	DEM
	Aspect	The degree of acceptance of sunlight	-	DEM
Neighborhood	The distance to the river	Human turbulence	m	Vector
	The distance to the road	Human turbulence	m	Vector
	The distance to the railway	Human turbulence	m	Vector
Meteorology	Average annual temperature	Multiyear average temperature	℃	CRU
	Annual precipitation	Multiyear precipitation	mm	CRU
Society	Population density	Human activity	people/km^2^	NASA

**Table 4:** Suitability constraints for changes in construction land, cultivated land, forest land and grassland.

Class	Slope	Aspect	The Distance to the Railway	The Distance to the River	The Distance to the Road
Artificial surfaces	1–2	3–8	1	1–3	1
Croplands	1–2	3–8	1–4	1–3	1–2
Forest	1–3	3–7	1–2	1–4	1–3
Grasslands	1–3	2–8	1–5	1–3	1–2

**Table 5:** Land Use Transfer Matrix of Selenga River Basin from 1992 to 2015. Unit: km^2^.

	1992	Artificial Surfaces	Croplands	Broadleaf Forest	Needleleaf Forest	Mixed Forest	Shrublands	Grasslands	Water	Barren
2015										
Artificial surfaces		82.47	15.95	0.5	33.3	31.92	41.15	168.66	3.74	13.7
Croplands		6.68	4505.34	51.48	757.11	8711.32	881.81	37,079.02	53.28	1020.64
Broadleaf forest		0	1.32	58.86	89.21	30.52	12.63	149.91	0.67	0.26
Needleleaf forest		1.25	5331.52	273.55	66662.51	4729.21	3465.25	10,325.64	40.01	144.16
Mixed forest		36.2	437.81	6649.26	46,452.4	10,900.39	5094.67	22,348.02	82.02	300
Shrublands		35.18	183.16	4120.54	16,203.67	12,828.81	16,871.99	39,469.03	262.96	1821.17
Grasslands		180.57	9633.53	69.11	2516.11	15,185.46	1872.5	93,250.9	210.01	11,261.12
Water		10.46	113.92	4.02	348.59	74.6	98.28	395.91	3521.66	81.6
Barren		0	0.78	0	0	0.36	0.66	2.46	11.11	17.32

## Data Availability

Not applicable.

## Appendix A

**Table A1:** Model estimates for cultivated land expansion driving forces.

Variable	1992–2000			2000–2009			2009–2015		
	Regression Coefficient β	Wald	EXP (β)	Regression Coefficient β	Wald	EXP (β)	Regression Coefficientβ	Wald	EXP (β)
Altitude	−0.412	29.707	0.662	−0.199	7.261	0.819	0.351	4.676	1.421
Slope 1	−2.365	0.000	0.000	−2.713	0.000	0.000	−2.039	0.000	0.000
Slope 2	−0.537	1.037	0.584	−0.252	0.454	0.777	−0.299	0.417	0.742
Slope 3	−0.199	0.144	0.820	0.067	0.026	1.070	0.162	0.131	1.176
Slope 4	0.146	0.076	1.157	0.177	0.177	1.193	0.128	0.081	1.136
Slope 5	0.420	0.549	1.521	0.281	0.406	1.324	−0.236	0.245	0.790
North slope	−0.958	2.191	0.384	0.019	0.016	1.020	0.132	0.580	1.142
Northeast slope	0.009	0.002	1.009	−0.048	0.086	0.953	0.171	0.861	1.186
East slope	−0.006	0.001	0.994	−0.227	2.190	0.797	0.275	2.432	1.317
Southeast Slope	0.265	2.154	1.303	0.032	0.036	1.033	0.089	0.171	1.093
South slope	−0.076	0.212	0.927	−0.008	0.002	0.992	0.184	0.668	1.202
Southwest slope	−0.150	0.910	0.861	−0.140	0.771	0.870	0.106	0.253	1.112
West slope	−0.253	2.396	0.776	−0.074	0.214	0.928	−0.167	0.668	0.846
Northwest slope	0.021	0.013	1.021	−0.026	0.023	0.975	0.254	1.769	1.289
Distance to river	0.187	25.593	1.205	0.036	0.903	1.037	−0.057	1.428	0.944
Distance to road	0.112	8.096	1.118	−0.115	8.405	1.121	0.059	1.576	1.061
Distance to railway	0.152	4.631	1.164	0.061	0.560	1.063	0.302	13.786	0.739
Average annual temperature	−0.695	117.658	0.499	−0.221	7.264	0.802	−0.125	0.374	0.121
Annual precipitation	0.397	50.513	1.488	0.180	7.632	0.835	0.235	7.513	1.265
Population density	0.056	2.371	1.058	0.071	2.900	1.073	0.017	3.141	1.017
Constant	1.599	8.850	4.949	0.373	0.778	1.452	−0.891	3.742	0.410

**Table A2:** Model estimates for cultivated land contraction driving forces.

Variable	1992–2000			2000–2009			2009–2015		
	Regression Coefficient β	Wald	EXP (β)	Regression Coefficient β	Wald	EXP (β)	Regression Coefficient β	Wald	EXP (β)
Altitude	0.218	9.459	0.804	−0.041	6.462	1.042	0.206	6.469	1.228
Slope 1	0.429	0.824	0.653	−2.027	0.000	0.000	1.461	0.966	4.309
Slope 2	0.831	1.028	0.795	0.815	3.856	2.259	0.905	4.926	2.471
Slope 3	0.827	1.015	0.787	0.811	3.883	2.250	0.351	0.772	1.421
Slope 4	0.573	0.922	0.273	0.559	1.826	1.750	−0.236	0.344	0.790
Slope 5	0.327	0.564	0.386	0.310	0.507	1.363	−0.349	0.682	0.706
North slope	−2.129	3.744	0.119	0.877	1.225	2.403	0.584	0.397	1.792
Northeast slope	−0.153	0.485	0.858	0.277	3.182	1.319	0.125	0.595	1.134
East slope	−0.050	0.062	0.951	0.365	6.437	1.441	0.356	5.367	1.428
Southeast Slope	−0.082	0.138	0.922	0.631	16.047	1.879	0.648	13.874	1.913
South slope	−0.174	0.752	0.840	0.889	34.726	2.433	1.150	42.769	3.158
Southwest slope	−0.215	1.288	0.806	1.054	54.336	2.869	1.167	50.140	3.212
West slope	−0.214	1.180	0.808	0.744	25.629	2.104	0.693	18.527	2.000
Northwest slope	−0.033	0.024	0.967	0.432	7.467	1.541	0.262	2.417	1.299
Distance to river	0.079	3.163	1.082	0.090	7.272	0.914	0.002	0.004	1.002
Distance to road	−0.130	8.822	0.878	−0.042	1.575	0.959	−0.086	4.890	0.918
Distance to railway	−0.195	6.621	0.823	−0.026	0.188	0.974	0.320	18.700	1.377
Average annual temperature	−0.112	13.318	0.894	0.363	1.024	0.054	0.616	4.117	0.540
Annual precipitation	−0.245	18.764	0.783	−0.042	0.707	1.043	0.309	15.473	1.362
Population density	−0.010	0.055	0.990	−0.009	0.075	0.991	0.145	12.305	1.156
Constant	−0.092	4.466	0.912	−0.250	0.351	0.779	−0.727	3.206	0.483

**Table A3:** Model estimates for Coniferous Forest land expansion driving forces.

Variable	1992–2000			2000–2009			2009–2015		
	Regression Coefficient β	Wald	EXP (β)	Regression Coefficient β	Wald	EXP (β)	Regression Coefficient β	Wald	EXP (β)
Altitude	−0.004	0.008	0.996	0.338	5.531	0.713	0.702	17.710	2.018
Slope 1	−2.193	0.000	0.000	−2.844	0.000	0.000	−1.918	0.000	0.000
Slope 2	0.864	9.470	2.373	0.825	2.702	2.281	0.627	2.961	1.872
Slope 3	0.368	1.822	1.445	0.694	1.950	2.002	−0.044	0.015	0.957
Slope 4	0.250	0.833	1.284	0.490	0.968	1.633	0.036	0.010	1.037
Slope 5	0.277	0.938	1.319	0.355	0.474	1.426	0.056	0.023	1.058
North slope	−0.138	1.399	0.871	−0.102	0.532	0.903	−0.012	0.007	0.988
Northeast slope	0.008	0.005	1.008	−0.011	0.005	0.989	0.035	0.053	1.035
East slope	−0.128	1.188	0.880	−0.254	2.926	0.775	0.180	1.510	1.197
Southeast Slope	−0.104	0.624	0.901	0.014	0.007	1.014	0.176	1.150	1.192
South slope	−0.127	1.058	0.881	0.224	2.318	1.252	0.228	2.164	1.256
Southwest slope	−0.075	0.447	0.928	−0.201	2.120	0.818	0.256	3.265	1.292
West slope	−0.153	1.605	0.858	−0.095	0.408	0.910	0.092	0.373	1.097
Northwest slope	0.087	0.455	1.091	−0.130	0.689	0.878	0.089	0.319	1.093
Distance to river	−0.118	8.191	0.889	0.075	5.118	1.078	−0.248	47.377	0.780
Distance to road	−0.312	2.301	0.732	0.184	23.729	0.832	0.333	80.515	0.717
Distance to railway	0.286	7.519	1.331	−0.088	4.226	0.915	0.501	82.937	1.651
Average annual temperature	−0.085	10.510	0.918	0.056	1.085	0.491	−0.743	3.259	0.223
Annual precipitation	0.110	14.875	1.117	0.259	44.788	1.296	0.875	94.958	2.399
Population density	−0.225	6.585	1.252	−0.087	1.563	0.917	−0.128	9.486	1.137
Constant	−0.150	0.280	0.861	−1.467	8.470	0.231	−1.566	18.122	0.209

**Table A4:** Model estimates for Coniferous Forest land contraction driving forces.

Variable	1992–2000			2000–2009			2009–2015		
	Regression Coefficient β	Wald	EXP (β)	Regression Coefficient β	Wald	EXP (β)	Regression Coefficient β	Wald	EXP (β)
Altitude	−0.336	41.240	0.715	−0.896	47.379	2.449	−0.074	0.716	0.928
Slope 1	−2.494	0.000	0.000	−9.039	0.000	0.000	−2.228	0.000	0.000
Slope 2	0.465	1.180	1.592	0.774	5.789	2.169	−1.033	9.526	0.356
Slope 3	0.383	0.835	1.467	0.215	0.474	1.240	−1.275	5.134	0.280
Slope 4	0.091	0.047	1.096	0.095	0.092	1.100	−1.656	14.662	0.191
Slope 5	0.001	0.000	1.001	0.089	0.075	1.093	−1.318	7.746	0.268
North slope	−0.109	0.461	0.896	−0.272	3.749	0.762	0.758	0.444	2.133
Northeast slope	0.066	0.152	1.068	−0.103	0.473	0.903	0.204	0.460	1.227
East slope	−0.022	0.019	0.978	0.273	3.832	1.314	0.058	0.035	1.059
Southeast Slope	0.224	1.651	1.251	0.298	3.681	1.347	0.124	0.135	1.132
South slope	0.389	5.828	1.475	0.374	6.574	1.454	0.931	11.179	2.537
Southwest slope	0.039	0.065	1.040	0.473	12.490	1.605	0.502	3.357	1.651
West slope	−0.028	0.029	0.973	0.176	1.502	1.192	0.132	0.182	1.141
Northwest slope	0.091	0.263	1.095	−0.006	0.002	0.994	0.000	0.000	1.000
Distance to river	−0.014	0.153	0.986	−0.251	17.190	0.778	0.027	0.192	1.028
Distance to road	−0.246	12.976	0.782	−0.356	31.019	0.701	0.003	0.002	1.003
Distance to railway	0.160	10.986	1.173	−1.133	61.395	3.105	−0.320	20.958	1.378
Average annual temperature	0.351	67.830	1.420	0.673	7.142	0.548	0.578	5.132	0.367
Annual precipitation	−0.137	38.097	0.872	−1.230	63.412	3.423	−0.319	19.754	1.375
Population density	−0.022	0.225	0.978	0.168	9.541	1.183	−0.094	6.052	0.910
Constant	−1.241	8.242	0.289	−0.568	3.035	0.566	−1.796	14.883	0.166

**Table A5:** Model estimates for Coniferous grassland expansion driving forces.

Variable	1992–2000			2000–2009			2009–2015		
	Regression Coefficient β	Wald	EXP (β)	Regression Coefficient β	Wald	EXP (β)	Regression Coefficient β	Wald	EXP (β)
Altitude	0.002	0.000	1.002	−0.180	20.207	0.835	−0.107	7.036	0.899
Slope 1	−1.712	0.000	0.000	−2.942	0.000	0.000	−2.876	0.000	0.000
Slope 2	−0.342	1.152	0.710	−1.335	9.604	0.263	−0.999	15.048	0.368
Slope 3	−0.041	0.017	0.960	−0.785	3.336	0.456	−0.776	9.281	0.460
Slope 4	0.407	1.627	1.502	−0.280	0.421	0.755	−0.554	4.616	0.575
Slope 5	0.373	1.200	1.452	−0.303	0.460	0.738	−0.609	4.987	0.544
North slope	0.048	0.005	1.049	0.920	1.479	2.508	0.558	0.963	1.748
Northeast slope	0.056	0.164	1.058	−0.136	1.011	0.873	−0.052	0.172	0.949
East slope	0.034	0.070	1.034	−0.168	1.850	0.845	−0.098	0.733	0.907
Southeast Slope	−0.116	0.709	0.890	−0.061	0.219	0.941	−0.229	3.643	0.795
South slope	−0.195	2.281	0.823	−0.115	0.885	0.892	−0.266	5.603	0.766
Southwest slope	−0.196	2.536	0.822	−0.152	1.713	0.859	−0.355	10.903	0.701
West slope	−0.102	0.621	0.903	−0.141	1.316	0.869	−0.351	9.490	0.704
Northwest slope	0.073	0.283	1.076	−0.164	1.518	0.849	−0.152	1.467	0.859
Distance to river	−0.115	17.344	0.891	−0.015	0.372	0.985	0.012	0.247	1.012
Distance to road	0.187	4.387	1.206	0.300	105.379	1.350	0.135	33.161	1.145
Distance to railway	−0.112	4.461	0.894	0.577	175.612	0.562	0.055	2.028	0.946
Average annual temperature	0.100	43.484	1.105	0.743	5.617	0.461	0.239	3.785	0.257
Annual precipitation	0.652	221.795	1.918	0.552	235.566	0.576	−0.383	97.793	0.682
Population density	−0.034	1.509	0.966	−0.081	10.794	0.922	−0.050	25.045	1.052
Constant	−0.094	0.084	0.910	1.109	6.379	3.031	0.436	2.665	1.547

**Table A6:** Model estimates for Coniferous grassland contraction driving forces.

Variable	1992–2000			2000–2009			2009–2015		
	Regression Coefficient β	Wald	EXP (β)	Regression Coefficient β	Wald	EXP (β)	Regression Coefficient β	Wald	EXP (β)
Altitude	0.052	0.857	1.053	−0.081	73.414	0.922	−0.390	3.373	0.677
Slope 1	−1.742	0.000	0.000	−3.084	0.000	0.000	−2.256	0.000	0.000
Slope 2	−0.354	1.276	0.702	−1.927	23.038	0.146	−0.978	7.723	0.376
Slope 3	−0.064	0.043	0.938	−1.714	18.414	0.180	−0.356	1.042	0.701
Slope 4	0.296	0.892	1.344	−1.272	9.995	0.280	0.074	0.045	1.077
Slope 5	0.220	0.426	1.246	−1.142	7.446	0.319	−0.249	0.448	0.780
North slope	0.254	0.133	1.290	1.208	2.917	3.345	0.169	0.065	1.184
Northeast slope	−0.120	0.690	0.887	−0.262	3.668	0.769	0.118	0.721	1.126
East slope	−0.169	1.591	0.844	−0.320	6.382	0.726	−0.304	5.407	0.738
Southeast Slope	−0.080	0.319	0.923	−0.428	9.965	0.652	−0.394	8.159	0.674
South slope	−0.226	2.898	0.798	−0.517	16.586	0.596	−0.526	16.397	0.591
Southwest slope	−0.225	3.180	0.798	−0.656	29.017	0.519	−0.696	31.564	0.498
West slope	−0.293	4.699	0.746	−0.423	11.063	0.655	−0.690	27.183	0.502
Northwest slope	−0.112	0.593	0.894	−0.300	4.861	0.741	−0.072	0.267	0.931
Distance to river	−0.062	4.280	0.940	0.046	2.977	1.047	0.025	0.848	1.025
Distance to road	0.031	0.974	1.032	0.155	31.819	1.167	−0.076	58.078	1.079
Distance to railway	0.181	1.120	1.198	−0.388	59.426	0.678	−0.420	81.066	0.657
Average annual temperature	−0.065	9.058	1.067	−0.487	3.291	0.854	0.539	5.732	0.386
Annual precipitation	−0.798	9.156	2.220	−0.604	153.922	0.547	−0.462	130.946	0.630
Population density	0.038	0.030	1.039	−0.001	0.001	0.999	0.013	0.278	1.013
Constant	−0.198	0.317	0.821	1.712	17.667	5.542	−0.200	0.307	0.819
